# Expression profiles of *BrMYB* transcription factors related to glucosinolate biosynthesis and stress response in eight subspecies of *Brassica rapa*

**DOI:** 10.1002/2211-5463.12231

**Published:** 2017-09-19

**Authors:** Mi‐Suk Seo, Mina Jin, Seong‐Han Sohn, Jung Sun Kim

**Affiliations:** ^1^ Genomics Division Department of Agricultural Bio‐Resources Rural Development Administration National Institute of Agricultural Sciences Wansan‐gu Jeonju Korea

**Keywords:** *Brassica rapa* subspecies, *BrMYB* transcription factors, DNA‐binding domain, desulfo, expression profiling, glucosinolate, stress defense mechanism

## Abstract

*Brassica rapa* is a polyploid species with phenotypically diverse cultivated subspecies. Glucosinolates (GSLs) are secondary metabolites that contribute to anticarcinogenic activity and plant defense in Brassicaceae. Previously, complete coding sequences of 13 *BrMYB* transcription factors (TFs) related to GSL biosynthesis were identified in the *B. rapa* genome. In the present study, we investigated GSL content and expression levels of these *BrMYB* TFs in 38 accessions belonging to eight subspecies of *B. rapa*. Twelve identified GSLs were detected and were classified into three chemical groups based on patterns of GSL content and expression profiles of the *BrMYB* TFs. GSL content and *BrMYB* TF expression levels differed among genotypes, including *B. rapa* subspecies *pekinensis*,* chinensis* and *rapa*. *BrMYB28.3*,* BrMYB51.1* and *BrMYB122.2* positively regulated GSL content in 38 accessions. Furthermore, expression levels of *BrMYB28*s and *BrMYB34.3* increased under most abiotic and biotic stress treatments. The three *BrMYB51* paralogs also showed drastically increased expression levels after infection with *Pectobacterium carotovorum*. The results of the present study improve our understanding of the functional diversity of these 13 *BrMYB* TFs during the evolution of polyploid *B. rapa*.

**Abbreviations**
Aa: amino acids
EST: expressed sequence tag
GSL: glucosinolate
TF: transcription factor

*Brassica rapa* belongs to the Brassicaceae family and has a number of subspecies with wide morphological variations. The subspecies are mainly distinguished by their leaf morphology (e.g. Chinese cabbage, pak choi and turnip types). The agricultural and nutritional properties of *Brassica* subspecies have resulted in their worldwide cultivation. The subspecies Chinese cabbage and pak choi are important vegetable sources and are also useful as fodder. Turnip rape subspecies are important oil crops in Asia and Europe. Furthermore, the *B. rapa* subspecies contain numerous nutritional products, including dietary fiber, vitamins and secondary metabolites, such as flavonoids, anthocyanins and glucosinolates (GSLs) 1. In addition to being an agriculturally important resource, *B. rapa* is also a model dicot plant for studies of polyploidy‐related genome evolution. *Brassica* and *Arabidopsis thaliana* diverged from a common ancestor and *Brassica* has undergone genome triplication recently 2, 3. The recent completion of the sequence of the *B. rapa* polyploid genome now allows for the identification of various gene families in its genome. In particular, hormone‐related genes (e.g. auxin‐related genes) and secondary metabolite‐related genes (e.g. GSL and anthocyanin) have been identified in the *B. rapa* genome by comparative genome analysis with *A. thaliana* 4, 5, 6, 7.

Glucosinolates are secondary metabolites that play an important role in plant defense mechanisms against environmental changes or stress. Recently, some GSLs and their breakdown products have attracted attention as a result of their anticarcinogenic and antioxidative activities in humans 8, 9. GSLs are classified into three groups: (a) aliphatic GSLs derived from the amino acids (aa) Met, Ala, Leu, Ile or Val; (b) indolic GSLs derived from Trp; and (c) aromatic GSLs derived from Phe or Tyr 10. GSLs are derived from aa and sugars via three primary biosynthetic steps: (a) aa chain‐elongation; (b) core GSL biosynthesis; and (c) side chain modification. Many studies on GSL biosynthesis have been performed using molecular biological approaches in *A. thaliana* 10, 11, 12. These have shown that some R2R3 MYB transcription factors (TFs) are regulators that control GSL biosynthesis. MYB28, MYB29 and MYB76 is a positive regulator of aliphatic GSLs in *Arabidopsis*, and high transcription levels of MYB28 result in the production of large amounts of aliphatic GSLs 13, 14. Furthermore, MYB34, MYB51 and MYB122 have been shown to regulate indolic GSL biosynthesis 12, 14. However, the identification of the complete regulation mechanism of GSL biosynthesis will require the expression analyses of additional genes and the use of different plant species. Recently, we reported the functional analysis of *BrMYB28* TFs using *Agrobacterium*‐mediated transformation in *B. rapa* 15. Our results suggested that GSL regulation in *B. rapa* involves a more complex GSL pathway than in *Arabidopsis* because of the complexity of the *B. rapa* genome following polyploid evolution. Therefore, understanding the regulation of GSL biosynthesis in *B. rapa* will provide useful information for research into secondary metabolites in other crop and plant species with polyploid genomes.

Glucosinolate content is known to vary in response to genetic and environmental factors in *Brassica* crops 16, 17, 18. Furthermore, GSL content also varies among plant tissues and developmental stage. *B. rapa* exhibits wide morphological phenotypic variation in features such as leaf and root shape, size and color 19. Therefore, it can be hypothesized that variations in GSL content in *B. rapa* may reflect a wide range of expression profiles of GSL biosynthesis‐related genes. Studies on GSLs in *B. rapa* have mostly been focused on variation of GSL content in many cultivars or on expression analyses of some genes related to GSL biosynthesis in *B. rapa* 20, 21, 22. There is a lack of expression profiles of genes related to GSL biosynthesis and GSL content variation in different *B. rapa* subspecies with wide morphological variation. A greater understanding of how expression of duplicated genes is involved in GSL content variation in *B. rapa* will provide greater insights into the evolution of duplicated gene expression and functional changes in this polyploid species.

We performed a comparative genome analysis aiming to identify MYB TFs that regulate GSL biosynthesis in *B. rapa* and compared their sequences with those of *Arabidopsis*. We also analyzed the expression levels of 13 *BrMYB* TFs and GSL content in 38 accessions of eight subspecies of *B. rapa*. MYB TFs are known to play important roles in the regulation of various biological processes, such as secondary metabolism, plant stress responses and plant development 23, 24. To better understand the functional diversity of MYB TFs related to GSL biosynthesis in *B. rapa*, we screened a microarray database aiming to identify *BrMYB* TFs that respond to abiotic and biotic stresses. Together, these results will be useful for understanding the functions of *BrMYB* TFs in GSL biosynthesis regulation and plant defense mechanisms in *B. rapa* and related polyploids.

## Materials and methods

### Plant material

Seeds from 38 accessions of eight *B. rapa* subspecies were obtained from the National Agrobiodiversity Center (Rural Development Administration, Republic of Korea). Seeds were sown in soil and plants were grown in a greenhouse under a 16‐h photoperiod at 24 °C. Fresh leaves of 6‐week‐old plants were harvested and used for analysis of GSL content and for real‐time PCR.

### Identification and analysis of MYB TFs in *B. rapa*

Genome information on GSL biosynthesis‐related TFs of *B. rapa* was obtained from the National Center for Biotechnology Information (NCBI, Bethesda, MD, USA) and the Brassica Database (http://brassicadb.org/brad). GSL biosynthesis‐related *BrMYB* TFs with complete coding sequence were analyzed. The R2R3 binding domain was predicted by smart analysis (http://smart.embl-heidelberg.de) and a multiple sequence alignment of 13 *BrMYB* TFs was performed using clustalw2 (http://www.ebi.ac.uk/Tools/msa/clustalw2). A phylogenetic tree was obtained using mega6 software (http://megasoftware.net) based on previously published MYB TFs related to the GSL biosynthesis pathway in *B. rapa* and *A. thaliana* 15. Expression profiles of *BrMYB* TFs in leaves of the cultivar Chiifu (*B. rapa* ssp. *pekinensis*) subjected to abiotic [cold (4 °C), salt (250 mm NaCl), drought (air‐dry) and ABA (100 μm)] and biotic (*Plasmodiophora brassicae* and *Pectobacterium carotovorum*) stress treatments were analyzed using the microarray and unigene database on the *B. rapa* Genome Project website (http://nabic.rda.go.kr). For additional detail on the abiotic stress experiments, 3‐week‐old plants (after germination and before the abiotic treatment) were used as controls. For the cold treatment, 3‐week‐old plants were placed in a growth chamber at 4 °C under continuous light. In the drought treatment, the plants were removed from pot together with soil and then air dried in a growth chamber. For salt and ABA treatments, the plants transferred to and grown in each of water containing 250 mm NaCl and 100 μm abscisic acid under continuous light. The plants for the abiotic experiments were treated to stress conditions for specific periods: 0.5, 3, 12, 24 and 48 h of treatment under cold, salt and ABA stress and 6, 12, 24 and 48 h of treatment under drought stress. For the biotic stress experiments and the infection of *P. brassicae*, the club root strains (race4) were homogenized in sterile distilled water in a blender and then the roots of the 1‐week‐old seedlings after germination were immersed into the suspension for 24 h. For the infection of *P. carotovorum*, the pathogens were grown for 24 h on PSA medium (10 g·L^−1^ peptone, 10 g·L^−1^ sucrose, 1 g·L^−1^ sodium‐glutamate and 15 g·L^−1^ agar). The bacterial pellets resuspended in sterile distilled water containing 0.9% NaCl (*D*_600_ = 0.2) and then top third upper part from the top of the leaves of 3‐week‐old plants was inoculated with the bacterial suspension. These experiments were performed with two biological replicates. Whole plants at abiotic stress treatments and tissues at biotic stress treatments frozen in liquid nitrogen at each time point and total RNA were isolated from each sample for microarray analysis. Detail experimental information with respect to construction of the microarray and the unigenes database is provided in Lee *et al*. 25.

### HPLC analysis for identification of GSL content

Desulfo (DS)‐GSLs were extracted in accordance with the procedure of Kim *et al*. 26. Fresh leaves of 6‐week‐old plants were freeze‐dried and 100 mg samples were used for protein extraction by boiling with 1.5 mL of 70% (v/v) methanol in a water bath. As an external standard, we used 0.5 mg of sinigrin (SNG) dissolved in 5 mL of ultrapure water. Crude extracts were loaded on Sephadex A25 columns and desulfated overnight using aryl sulfatase (EC3.1.6.1) prior to HPLC. DS‐GSLs were analyzed using a 1200 series HPLC system (Agilent Technologies, Santa Clara, CA, USA) equipped with an Inertsil ODS‐3 column (150 × 3.0 mm inner diameter, particle size 3 μm; GL Science, Tokyo, Japan). The HPLC analysis was carried out using a flow rate of 0.4 mL·min^−1^ at a column oven temperature of 35 °C and a wavelength of 227 nm. The individual GSLs were quantified by comparison with the external standard SNG and the values for total GSLs were obtained by summing the values of the identified individual GSLs (Table 1). Data were recorded for individual, total aliphatic, total indolic and total GSL content samples. The experiment was performed as three biological replicates and the data obtained were used to calculate means.

**Table 1:** GSLs identified in 38 accessions of *Brassica rapa*

Number	Systematic name	Trivial name	Abbreviation
Aliphatic			
1	2‐Hydroxy‐3‐butenyl	Progoitrin	PRO
2	4‐Methylsulfinylbutyl	Glucoraphanin	GRA
3	2‐Propenyl	Sinigrin	SNG
4	5‐Methylsulfinylpentyl	Glucoalyssin	GAL
5	2‐Hydroxy‐4‐pentenyl	Gluconapoleiferin	GNL
6	3‐Butenyl	Gluconapin	GNA
7	1‐Methylpropyl	Glucocochlearin	GCC
8	4‐Pentenyl	Glucobrassicanapin	GBN
Indolic			
9	3‐Indolylmethyl	Glucobrassicin	GBS
10	4‐Methoxy‐3‐indolylmethyl	4‐Methoxyglucobrassicin	4‐MOGBS
11	1‐Methoxy‐3‐indolylmethyl	Neoglucobrassicin	NGBS
Aromatic			
12	2‐Phenylethyl	Gluconasturtiin	GNT

### RNA extraction and real‐time PCR analysis

Total RNA was isolated from whole plant leaves using a Hybrid‐R kit (GeneAll, Seoul, Korea) and treated with RNase‐free DNase I (Takara, Tokyo, Japan) to eliminate contaminating genomic DNA. Approximately 2 μg of total RNA was reverse transcribed into cDNA with oligo‐dT primers using a first‐strand cDNA synthesis kit (Gendepot, Katy, TX, USA). The synthesized cDNAs were diluted ten‐fold in sterilized water and a real‐time PCR was performed using 2 μL of diluted cDNA in 20 μL of SYBR^®^ Green mix (GeneAll). The primers for member‐specific detection of expression of *BrMYB* TFs were designed for the 3′‐terminal region. The *Bractin* gene primer was used as a control for all expression analyses. The gene‐specific primers used for real‐time PCR analysis are shown in Table S1. We used the thermal cycler conditions recommended by the manufacturer: 40 cycles of 95 °C for 10 s, 55 °C for 20 s and 72 °C for 30 s. All experiments were performed as three biological and technical replicates and the data obtained were used to calculate means.

## Results

### Characterization of *BrMYB* TFs related to GSL biosynthesis

**Figure 1:**
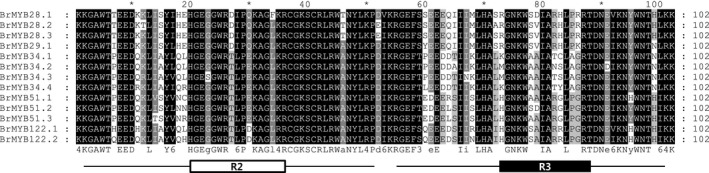
ClustalW aa sequence alignment of the R2R3‐MYB domain in GSL biosynthetic TFs of 13 *Brassica rapa* accessions. The shading of the alignment represents different degrees of conservation among sequences. The R2 and R3 binding domains are boxed in white and black, respectively.

In total, we identified 13 orthologous copies with complete coding sequences corresponding to five MYB TFs, a consequence of genome triplication in *B. rapa*, by comparative genomic analysis with *A. thaliana* (Table 2). Three orthologous copies of MYB28 TF were found in the *B. rapa* genome and all shared more than 80% sequence identify with *Arabidopsis*. Only one copy of MYB29 TF, *BrMYB29*, was identified in the *B. rapa* genome and this showed the lowest identity (76%) to the corresponding *Arabidopsis* sequence. Four copies of MYB34 were found and all shared more than 80% sequence identity with *Arabidopsis*. Three *BrMYB51* TFs were identified: *BrMYB51.1* and *BrMYB51.3* showed 78% sequence identity to *Arabidopsi*s; *BrMYB51.2* showed 80% sequence identity. Two copies of MYB122 TF were found and showed 81% and 82% sequence identity to *Arabidopsis*. The 13 *BrMYB* TFs had variations in orthologous and paralogous gene lengths. Additionally, the 13 *BrMYB* TFs paralogs were present on different bacterial artificial chromosome clones, indicating variation in chromosomal positions. Amino acid sequence alignments of the 13 *BrMYB* TFs showed that they were highly conserved at the orthologous and paralogous levels in the R2R3‐MYB domain (Fig. 1). All 13 *BrMYB* TFs had two typical R2R3 MYB‐DNA‐binding domains (DBDs), were 102 aa in length, and exhibited more than 90% sequence identity to each other. These results indicate variation in gene length (bp) and nonsynonymous aa sequences caused by polymorphisms in the C‐terminal region.

**Table 2:** DNA sequence summary of the *BrMYB* TFs related to GSL biosynthesis in *Brassica rapa*

Arabidopsis gene ID	*B. rapa* gene name	*B. rapa* gene ID	Length			Chromosome	EST clones	CDS identity to *Arabidopsis* (%)
			Gene (bp)	CDS (bp)	Protein (aa)			
AT5G61420	*BrMYB28.1*	Bra012961	1349	1065	354	A03	KBLS‐095C01	82
	*BrMYB28.2*	Bra035929	1370	1074	357	A09	KBFL‐120H07	84
	*BrMYB28.3*	Bra029311	1617	1119	372	A02	KFFB‐103G11	81
AT5G07690	*BrMYB29.1*	Bra005949	1410	993	330	A03	KBCD‐052H08	76
AT5G60890	*BrMYB34.1*	Bra035954	1148	909	302	A09	KBCG‐059A10	84
	*BrMYB34.2*	Bra013000	1390	951	316	A03	KHRT‐17B07	82
	*BrMYB34.3*	Bra029349	1172	843	280	A02	KHCT‐21C09	81
	*BrMYB34.4*	Bra029350	6952	930	309	A02	KBLS‐091A11	81
AT1G18570	*BrMYB51.1*	Bra031035	1251	963	320	A09	KHLD‐17B11	78
	*BrMYB51.2*	Bra016553	1538	1002	333	A08	KHCT‐02G03	80
	*BrMYB51.3*	Bra025666	1278	1026	341	A06	KHRT‐27H04	78
AT1G74080	*BrMYB122.1*	Bra015939	1455	981	319	A07	–	81
	*BrMYB122.2*	Bra008131	1929	1005	334	A02	–	82

**Table 3:** Total and individual GSL level (μmol·g^‐−1^ dw) in leaves of 38 accessions belonging to eight subspeices of *Brassica rapa*

Genotype (subspecies)	Accession number	Aliphatic								Indolic			Aromatic	Total GSLs
		PRO	GRA	SNG	GAL	GNL	GNA	GCC	GBN	GBS	4‐MOGBS	NGBS	GNT	
Caixin (ssp. *parachinensis*)														
Mean	2	2.01	0.07	0.03	0.74	0.16	5.16	2.84	2.67	0.50	0.20	0.21	0.20	14.80
Range		1.10–2.93	0.02–0.13	0.01–0.05	0.58–0.89	0.11–0.17	1.56–8.77	0.00–5.67	1.48–3.86	0.50–0.51	0.13–0.28	0.13–0.29	0.15–0.25	5.99–23.6
Chinese cabbage (ssp. *pekinensi*s)														
Mean	19	0.87	0.07	0.03	0.60	0.16	2.89	0.36	2.41	2.40	1.54	0.94	0.44	12.75
Range		0.13–2.59	0.00–0.33	0.00–0.10	0.00–2.45	0.00–0.63	0.02–28.02	0.00–2.27	0.12–11.28	0.50–4.08	0.60–3.36	0.07–2.47	0.02–1.30	5.41–48.17
Mizuna (ssp. *nipposinica*)														
Mean	1	0.20	0.69	0.03	0.58	0.00	25.56	0.46	2.39	2.34	0.51	0.47	0.15	33.38
Pak choi (ssp. *chinensis*)														
Mean	5	0.37	0.03	0.04	0.23	0.04	3.21	0.14	1.93	1.02	0.55	0.28	0.35	9.38
Range		0.07–1.02	0.00–0.07	0.00–0.15	0.04–0.44	0.00–0.19	0.84–5.08	0.00–0.33	0.83–2.9	0.58–1.67	0.41–0.88	0.08–0.64	0.2–0.49	4.93–11.5
Pak choi (ssp. *narinosa*)														
Mean	1	0.37	0.00	0.00	0.11	0.12	0.89	0.00	0.89	0.77	0.43	0.52	0.25	4.42
Summer oil (ssp. *dichotoma*)														
Mean	2	0.19	0.02	0.01	0.06	0.25	16.83	0.29	1.73	0.17	0.37	0.12	0.31	20.35
Range		0.05–0.34	0.00–0.03	0.00–0.02	0.04–0.07	0.00–0.49	0.33–33.34	0.13–0.45	1.09–2.37	0.10–0.24	0.23–0.51	0.09–0.15	0.26–0.37	4.72–35.98
Turnip (ssp. *rapa*)														
Mean	6	1.17	0.27	0.02	0.56	0.07	20.07	2.69	7.08	1.77	0.40	0.37	0.50	34.98
Range		0.20–2.36	0.00–0.65	0.00–0.03	0.15–1.47	0.00–0.2	5.83–35.05	0.00–12.84	0.74–14.93	1.38–2.02	0.32–0.57	0.11–0.73	0.15–0.68	11.20–49.95
Winter oil (ssp. *trilocularis*)														
Mean	2	2.65	0.00	0.06	0.00	0.00	45.68	0.01	3.24	0.56	0.30	0.45	0.56	53.51
Range		0.03–5.28	0.00	0.08–0.04	0.00	0.00	37.04–54.32	0.00–0.03	2.66–3.82	0.36–0.76	0.29–0.31	0.31–0.6	0.32–0.79	47.46–59.56

The means indicate the average of subspecies with the average of triplicate measurements in three independent biological replicates.

The two genotypes of mizuna (ssp. *nipposinica*) and pak choi (ssp. *narinosa*) underwent analyses of GSL content in only one accession.

**Table 4:** Correlation analysis of relative expression levels of *BrMYB* TFs related to GSL biosynthesis and GSL content in 38 accessions of *Brassica rapa*

Gene	PRO	GRA	SNG	GAL	GNL	GNA	GCC	GBN	Aliphatic GSLs	GBS	4‐MOGBS	NGBS	Indolic GSLs	GNT	Total GSLs
*BrMYB28.1*	−0.066	−0.102	−0.122	−0.034	−0.128	−0.098	−0.046	−0.120	−0.124	0.025	0.214	−0.103	0.051	−0.072	−0.122
*BrMYB28.2*	0.158	−0.215	0.166	−0.238	−0.052	−0.046	−0.073	−0.158	−0.083	0.173	0.341*	−0.065	0.194	−0.025	−0.057
*BrMYB28.3*	0.470**	−0.207	0.112	−0.012	−0.005	0.073	0.384*	0.288	0.203	−0.234	−0.210	−0.252	−0.271	0.110	0.175
*BrMYB29.1*	0.030	0.210	0.049	−0.022	−0.136	0.119	−0.120	0.152	0.120	0.043	−0.036	−0.154	−0.037	0.149	0.122
*BrMYB34.1*	−0.050	−0.050	−0.125	−0.219	0.143	−0.228	0.241	−0.008	−0.175	0.132	0.158	−0.013	0.122	−0.077	−0.166
*BrMYB34.2*	−0.088	−0.145	0.026	−0.191	0.065	−0.166	−0.063	−0.170	−0.200	0.011	0.049	−0.065	0.004	−0.199	−0.209
*BrMYB34.3*	−0.192	−0.216	−0.070	−0.193	−0.131	−0.281	−0.173	−0.112	−0.312	0.214	0.151	−0.090	0.138	−0.203	−0.305
*BrMYB34.4*	−0.062	0.097	0.016	0.204	−0.147	0.278	−0.029	0.217	0.283	−0.122	−0.118	−0.051	−0.121	0.104	0.277
*BrMYB51.1*	−0.156	0.080	−0.182	0.139	−0.186	−0.203	−0.075	−0.196	−0.233	0.446**	0.255	0.378*	0.434**	−0.066	−0.185
*BrMYB51.2*	0.038	−0.011	0.129	−0.020	0.205	−0.129	−0.080	−0.097	−0.139	−0.175	−0.051	0.154	−0.063	−0.135	−0.152
*BrMYB51.3*	−0.156	−0.105	−0.178	−0.118	−0.170	0.028	0.135	−0.132	−0.002	−0.025	0.150	−0.199	−0.017	−0.099	−0.006
*BrMYB122.1*	−0.169	−0.113	0.082	−0.178	−0.188	−0.049	−0.080	−0.037	−0.081	−0.107	−0.082	−0.103	−0.115	0.013	−0.097
*BrMYB122.2*	−0.064	−0.107	0.585**	−0.105	−0.191	−0.184	0.065	−0.159	−0.192	−0.114	−0.058	−0.170	−0.130	−0.148	−0.213

Significantly correlated: *P < 0.05, **P < 0.01, respectively.

### Profile of GSL content in 38 *B. rapa* accessions

**Figure 2:**
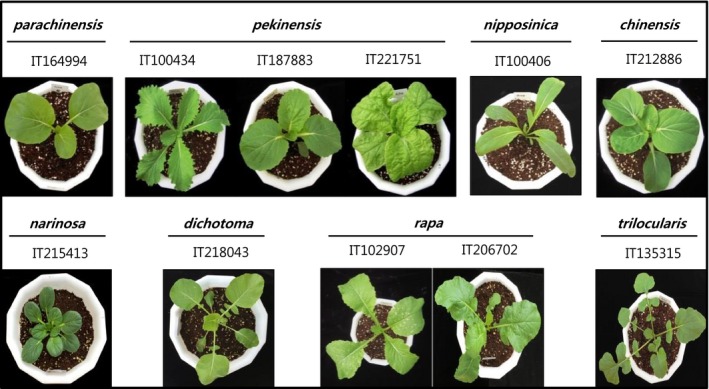
Phenotypes of eight subspecies of *Brassica rapa* used in the present study. Subspecies of each accession were extracted from the passport data of RDA Genebank. Genotypes for subspecies follow the nomenclature of Zhao *et al*. 45. Genotypes for subspecies were confirmed by a field test in 2011.

We examined the regulation of GSL biosynthesis in 38 accessions of eight subspecies of *B. rapa* that exhibited morphological variation in leaf shape (Fig. 2). Lou *et al*. 27 performed an analysis of quantitative trait loci that controlled various phenotypic characteristics, including leaf morphology, in *B. rapa* subspecies; they devised a classification system for leaf edge shape comprising: (a) entire; (b) slightly serrated; (c) intermediately serrated; and (d) very serrated. We used this system to classify the eight subspecies analyzed: (a) *parachinensis*,* chinensis*,* narinosa* were entire; (b) *pekinensis* was slightly serrated; (c) *rapa* was intermediately serrated; and (d) *dichotoma* and *trilocularis* were very serrated. The *B. rapa* ssp. *nipponsinica* IT100406 characteristically had elongated leaves within the non‐serrated category.

**Figure 3:**
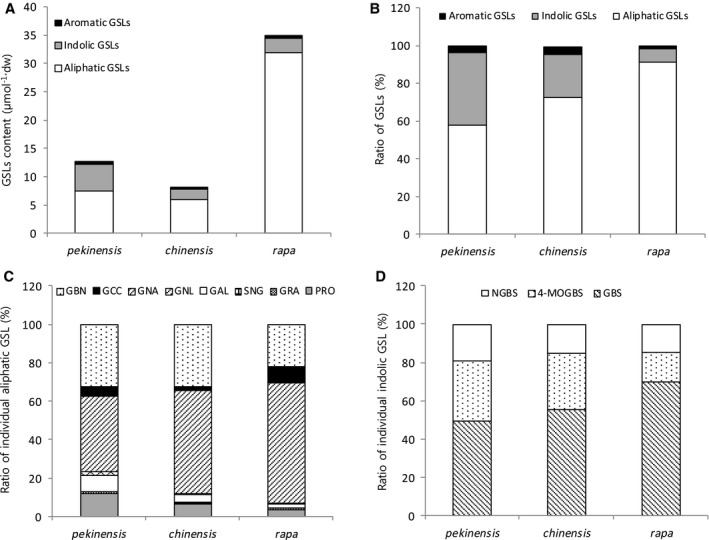
Identification of individual GSL composition in the three subspecies of *Brassica rapa*. (A) Content of aliphatic, indolic and aromatic GSLs comprising total GSL content for three subspecies. (B) Ratio of aliphatic, indolic and aromatic GSLs in total GSL content of three subspecies. (C) Ratio of individual GSL in aliphatic GSLs of three subspecies. (D) Ratio of individual GSL in indolic GSLs of three subspecies. GRA, glucoraphanin.

Twelve GSLs belonging to the aliphatic, indolic, and aromatic classes were detected in the 38 accessions (Table 1). The GSL contents of leaves of all 38 accessions were analyzed by HPLC (Table 3). Total GSL content ranged from 4.42 (pak choi, ssp. *narinosa*) to 59.56 μmol·g^−1^ dw (ssp. *trilocularis*). *B. rapa* ssp. *rapa* turnip types had intermediately serrated leaf edges and *B. rapa* ssp. *dichotoma* and *trilocularis* belonging to the oil types showed very serrated leaf shapes and were found to exhibit the highest mean total GSLs (ranging from 20.35 to 53.51 μmol·g^−1^ dw). By contrast, the lowest mean total GSLs (9.38 and 4.42 μmol·g^−1^ dw) were found in *B. rapa* ssp. *chinensis* and *narinosa* pak choi type that had an entire leaf edge phenotype. The 19 accessions of *B. rapa* ssp. *pekinensis* belonging to the Chinese cabbage type showed mean total GSLs of 12.75 μmol·g^−1^ dw, ranging from 5.41 to 48.17 μmol·g^−1^ dw. In the present study, we focused on the three main groups that distinguish morphotypes, such as *B. rapa* ssp. *pekinensis* (19) for Chinese cabbage type; *B. rapa* ssp. *chinensis* (five) for pak choi type; and *B. rapa* ssp. *rapa* (six) for turnip type (Fig. 3). A higher total GSL content (34.98 μmol·g^−1^ dw) and a higher aliphatic GSL content were found in *B. rapa* ssp. *rapa* compared to the other two subspecies (Fig. 3A). Aliphatic GSLs were found in high ratios in all three subspecies; in particular, *B*. *rapa* ssp. *rapa* exhibited a high aliphatic GSL ratio and a low ratio of indolic and aromatic GSLs compared to the other two subspecies (Fig. 3B). With regard to individual GSL ratios, gluconapin (GNA; aliphatic GSL) and glucobrassicin (GBS; indolic GSL) were detected at the highest levels in all three subspecies (Fig. 3C,D).

### Expression profile of *BrMYB* TFs related to GSL biosynthesis in the 38 accessions

**Figure 4:**
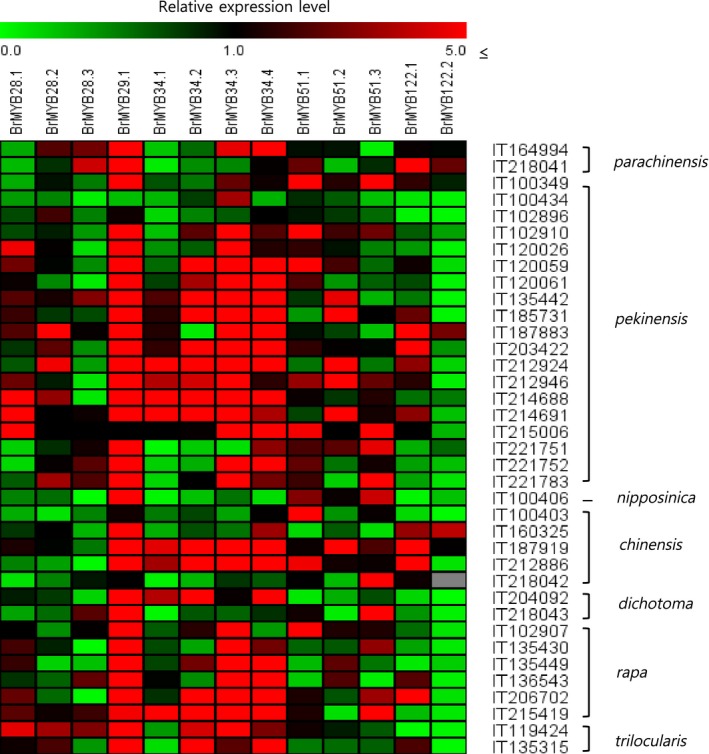
Relative expression analysis of *BrMYB* TFs related to GSL biosynthesis in 38 accessions of *Brassica rapa*. The *Bractin* gene was used as the quantitative control. Values indicated the average of three biological replicates. The relative expression was calculated as the fold increase relative to *ssp. narinosa* IT215413 with the lowest total GSL content.

To investigate the regulatory control of GSL biosynthesis in *B. rapa*, the expression levels of the 13 *BrMYB* TFs were analyzed by real‐time PCR in all 38 accessions (Fig. 4). The relative expression was calculated as the fold increase relative to *B. rapa* ssp. *narinosa* (IT215413), which had the lowest total GSL content. The relative expression level of *BrMYB29.1* was high and that of *BrMYB122.2* was low in most of the accessions. Relative expression of *BrMYB29.1*,* BrMYB34.3* and *BrMYB34.4* was higher in *B. rapa* ssp. *pekinensis*, whereas *BrMYB28.3* was lower in all but three accessions. In *B. rapa* ssp. *chinensis*, which has a low total GSL content, the relative expression of all three paralogs of *BrMYB28* was low in all five accessions. In addition, in *B. rapa* ssp. *rapa*, which had a high total GSL content, the relative expression of *BrMYB34.3* was higher in all six accessions, whereas that of *BrMYB34.4* was higher in all accessions except IT102907 and, in addition, *BrMYB51.1* was lower in all accessions except IT102907 (Fig. 4).

### Relationship between *BrMYB* TF expression and GSL content

We investigated the relationship between expression of the 13 *BrMYB* TFs and GSL content by correlation analyses in all 38 accessions (Table 4). *BrMYB28.2* was found to be positively correlated with 4‐methoxyglucobrassicin (4‐MOGBS; *P* < 0.05), an indolic GSL, whereas *BrMYB28.3* was significantly and positively correlated with the aliphatic GSLs progoitrin (PRO; *P* < 0.01) and glucocochlearin (GCC; *P* < 0.05). *BrMYB51.1* was also positively correlated with total indolic GSLs (*P* < 0.01), GBS (*P* < 0.01) and neoglucobrassicin (NGBS; *P* < 0.05). *BrMYB122.2* was significantly and positively correlated with SNG (*P* < 0.01), an aliphatic GSL, although low mRNA transcript levels were detected in most of the 38 accessions. The correlation of *BrMYB* TF expression and GSL content was also analyzed for individual subspecies (Tables S2, S3 and S4). As found for the analysis of all accessions, *BrMYB28.3*,* BrMYB51.1* and *BrMYB122.2* were positively correlated with GSL content in *B. rapa* ssp. *pekinensis* and *chinensis* (Tables S2 and S3). In addition, *BrMYB51.3* was positively correlated with PRO and gluconapoleiferin (GNL; i.e. aliphatic GSLs) in *B. rapa* ssp. *chinensis* (Table S3). The indolic GSL 4‐MOGBS was positively correlated with various *BrMYB* TFs, including *BrMYB29*,* BrMYB51.2* and two *BrMYB34* paralogs in *B. rapa* ssp. *chinensis*. In *B. rapa* ssp. *rapa*, four *BrMYB34* paralogs were found to be positively correlated with the aliphatic GSLs GCC, glucobrassicanapin (GBN) and glucoalyssin (GAL; Table S4). Taken together, these results indicate that the 13 *BrMYB* TFs function as positive regulators in different parts of the GSL biosynthesis pathway in *B. rapa*.

### Expression profile of *BrMYB* TFs in different developmental stages

**Figure 5:**
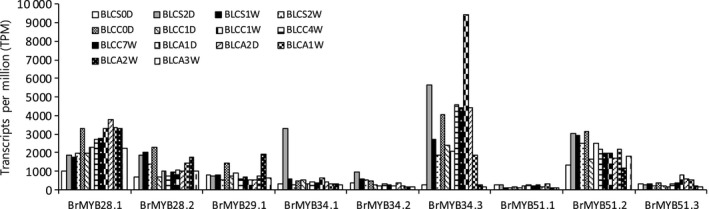
Expression analysis of nine *BrMYB* TFs related to GSL biosynthesis at different developmental stages in *Brassica rapa*. BLCS0D, seeds, mature; BLCS2D, seedling (2 days old); BLCS1W, whole plant, 1 week old vegetative stage (7 days old); BLCS2W, whole plant, 2 weeks old vegetative stage (14 days old); BLCC0D, whole plant, 3 weeks old vegetative stage (21 days old); BLCC1D, whole plant, 1 day after light‐chilled at 4 °C (22 days old); BLCC1W, whole plant, 1 week after light‐chilled at 4 °C (28 days old); BLCC4W, whole plant, 4 weeks after light‐chilled at 4 °C (56 days old); BLCC7W, whole plant, 7 weeks after light‐chilled at 4 °C (70 days old); BLCA1D, whole plant, 1 day after greenhouse growth (71 days old); BLCA2D, whole plant, 2 days after green house growth (72 days old); BLCA1W, whole plant, 1 week after green house growth (77 days old; BLCA2W, whole plant, 2 weeks after greenhouse growth (84 days old); BLCA3W, whole plant, 3 weeks after greenhouse growth (91 days old). LCS, vegetative stage; LCC, chilling treatment (vernalization); LCA, growth stage after chilling treatment (reproductive stage).

Expressed sequence tag (ESTs) were identified for all *BrMYB* TFs except the *BrMYB122*s in the Brassica database (http://nabic.rda.go.kr; Table 2). Expression profiles of these 11 *BrMYB* TFs were analyzed in different developmental stages of *B. rapa* using a microarray database 25 (Fig. 5). Two of the TFs, *BrMYB28.3* and *BrMYB34.4*, were not expressed, despite the presence of two ESTs extracted from the database: KFFB‐103G11 and KBLS‐091A11. Expression of the nine remaining *BrMYB* TFs was observed in most developmental stages. *BrMYB28.2*,* BrMYB34.2* and *BrMYB51.2* exhibited higher expression in vegetative stages (BLCS2D–BLCC0D) compared to reproductive stages (BLCA1D–BLCA3W), whereas *BrMYB28.1*,* BrMYB34.3* and *BrMYB51.3* were more highly expressed during the reproductive stages. *BrMYB34.3* was expressed at higher levels than the other *BrMYB* TFs in various developmental stages, with a maximum expression level of 71 days in a whole plant (BLCA1D). These results indicate that expression of the *BrMYB* TF paralogs occurred in different patterns and at different levels depending on the plant developmental (life history) stage.

### Expression profiles of *BrMYB TFs* under abiotic stresses

**Figure 6:**
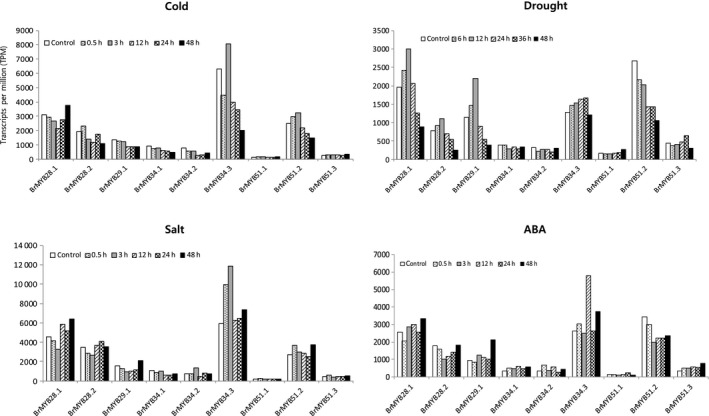
Microarray expression analysis at different abiotic stresses of nine *BrMYB* TFs related to GSL biosynthesis of *Brassica rapa*. Control, 3‐week‐old whole plant; cold, 4 °C cold treatment; drought, air‐dry; salt, 250 mm NaCl; ABA, 100 μm abscisic acid.

The effects of four abiotic stress treatments, namely cold, drought, salt and ABA, on the expression of *BrMYB* TFs were analyzed (Fig. 6). A cold stress treatment of 4 °C caused an initial decrease in the expression of *BrMYB28.1* but a subsequent increase by 24 h. Expression of *BrMYB28.2* and of *BrMYB34.3* increased under cold stress and was higher than the control at 0.5 and 3 h, respectively. *BrMYB51.2* expression increased until 3 h after treatment, although it decreased thereafter. Under drought stress conditions, expression of *BrMYB28.1*,*2* and *BrMYB29.1* significantly increased until 12 h of treatment, and gradually decreased thereafter. Expression *BrMYB34.3* and *BrMYB51.3* increased to 36 h, plateaued, and then decreased at 48 h. *BrMYB28.1* and *BrMYB28.2* exhibited identical responses, with an increased expression from 12 to 48 h under salt stress conditions. *BrMYB34.3* showed a higher expression level compared to the control during the salt stress treatment. After ABA treatment, expression of *BrMYB34.1* and *BrMYB51.3* increased compared to the control. After ABA stress, expression of *BrMYB28.1* and *BrMYB29.1* increased at 3 h post‐treatment. The variation in expression patterns observed for the nine *BrMYB* TFs related to GSL biosynthesis during the four abiotic stress treatments reflects their potential as regulators (effectors) for abiotic stress resistance. In particular, the increased expression of *BrMYB28s* and *BrMYB34.3* in the different stress treatments highlights the importance of these TFs in plant stress responses.

### Expression profile of *BrMYB* TFs under two biotic stresses

**Figure 7:**
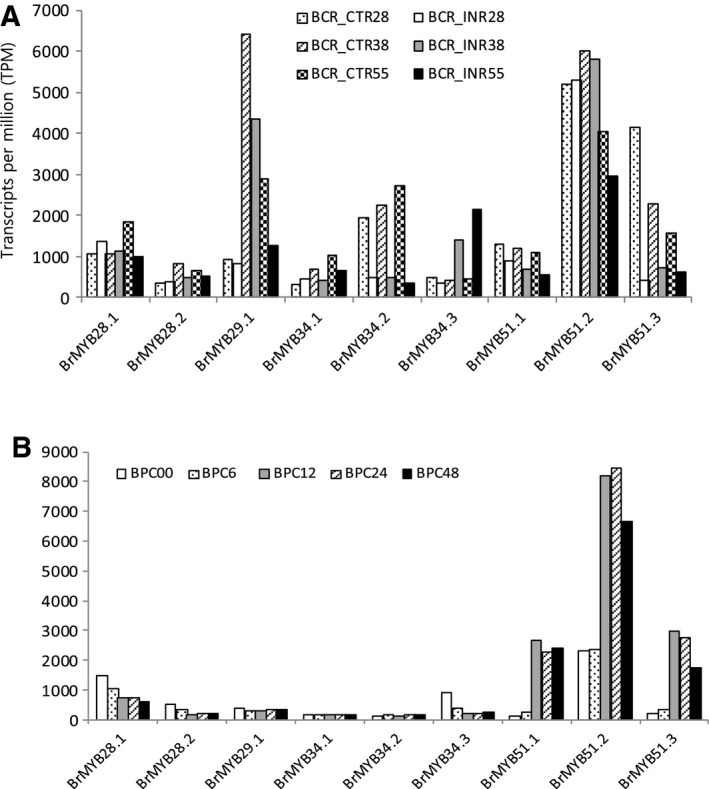
Microarray analysis of effects of two biotic stresses on the expression of nine *BrMYB* TFs related to GSL biosynthesis in *Brassica rapa*. (A) Expression changes after infection with *Plasmodiophora brassicae* (BCR_CTR28, non‐infected 28‐day‐old roots; BCR_INR28, 28‐day‐old roots infected by *P. brassicae*; BCR_CTR38, non‐infected 38‐day‐old roots; BCR_INR38, infected 38‐day‐old roots; BCR_CTR55, non‐infected 55‐day‐old roots; BCR_INR55, infected 55‐day‐old roots). (B) Expression changes after infection of *Pectobacterium carotovorum* (BPC00, non‐infected reference, leaf, one‐third upper part from the top; BPC6–48, 6–48 h post infection, non‐necrotic).

We also analyzed changes in expression level of nine *BrMYB* TFs under two biotic stress conditions (Fig. 7). *BrMYB28.1*,*2*,* BrMYB34.1* and *BrMYB51.2* showed increased expression in roots of 28‐day‐old plants infected with *P. brassicae*, which causes clubroot disease in *Brassica* (Fig. 7A). Expression of *BrMYB34.3* was significantly increased at 38 and 55 days after infection. After infection with *P. carotovorum*, which causes soft rot disease in *Brassica*, all three *BrMYB51* paralogs exhibited significantly increased expression (Fig. 7B). These results suggest that some *BrMYB* TFs may potentially regulate resistance to biotic stress, including infection responses.

## Discussion

MYB TFs are one of the largest gene families of plant TFs and are known to perform many functions in plant biological processes 28, 29. MYB TFs are classified by a conserved DNA‐DBD, which contains R1, R2 or R3 repeats; most plant MYB TFs are classified as R2R3‐MYB with two repeats in the N‐terminal region 30. R2R3 MYB TFs regulate plant responses to several signaling molecules 31. In the present study, all 13 *BrMYB* TFs were found to have highly conserved aa sequences in the R2 and R3 repeats of the DBD. However, polymorphisms within the C‐terminal region between paralogous genes led to structural divergence. Furthermore, the chromosomal locations of the paralogous genes differed. These results indicate that the polyploid evolution of the *Brassica* genome led to sequence divergence, such as indels that produced rearrangement of the *BrMYB* TFs related to GSL biosynthesis. Sequence divergence and chromosomal translocation of the triplicate paralogous genes has been reported in many *Brassica* species 32, 33, 34. Consequently, the duplication of genes by polyploidy speciation in *Brassica* species has led to functional diversity among the different species 35.

In the present study, we investigated the patterns of expression of 13 *BrMYB* TFs related to GSL content in eight subspecies of *B. rapa*. Twelve GSLs belonging to three chemical classes were detected in the 38 accessions of *B. rapa*. The total GSL contents of *B. rapa* ssp. *parachinensis*,* chinensis* and *narinosa*, which have leaf morphologies classified as entire, were found to be low. The *B. rapa* ssp. *dichotoma* and *rapa*, which have intermediately serrated leaves, were found to have a higher total GSL content than subspecies with entire or slightly serrated leaf edges. The highest total GSL content was found in *B. rapa* ssp. *trilocularis*, which has a very serrated leaf shape. Thus, we suggest that the morphogenesis of leaf edge shape may be regulated by the GSL biosynthesis pathway.

In the GSL profiles of different accessions, aliphatic GSLs were found to be most abundant, and GNA was the most abundant GSL recorded. Previous studies have reported the same GSL pattern in leaves 22, 36. These results indicate that the GSL content profiles in these 38 accessions are representative of *B. rapa* in general. Most research on the GSL content of *Brassica* genus has focused on differences among cultivars or the effects of environmental factors 18, 37, 38. The varieties were significantly different for total GSL content as well because, for all the individual GSLs, content in *B. rapa* varieties was dependent upon the crops and genotype 20. In the present study, significant differences in GSL content were identified in three main genotypes, including *pekinensis* of the Chinese cabbage type, *chinensis* of the pak choi type and *rapa* of the turnip type. Turnip type accessions were found to have the highest GSL contents, with increased aliphatic GSLs. The content of aliphatic GSLs is influenced by genetic and morphological variation in *B. rapa*. Therefore, indolic GSLs are influenced by environmental factors, whereas they are less affected by genetic factors than aliphatic GSLs 17.

Paralogous genes showed different expression patterns among accessions with the same ancestral gene. These results indicate that gene duplication through genome polyploidy in *B. rapa* has led to functional diversity and alteration of expression patterns, as reflected by genotype‐specific variation. *BrMYB28.3*,* BrMYB51.1* and *BrMYB122.2* were identified as positive regulators of GSL content in *B. rapa* ssp. *pekinensis* and *chinensis*. In the case of *B. rapa* ssp. *rapa* with drastically increased aliphatic GSLs, the four paralogous genes of *BrMYB34* and *BrMYB28.3* showed positive correlations with aliphatic GSLs. The distinct correlation patterns of *B. rapa* ssp. *rapa* might be explained by the individual GSL content. Some R2R3 MYB TFs were recently shown to regulate GSL biosynthesis in *Arabidopsis*, with MYB28 as a positive regulator of aliphatic GSLs, and MYB34, MYB51 and MYB122 as positive regulators of indolic GSLs 12, 13, 14. Similarly, we found that the *BrMYB28.3* was positively correlated with the aliphatic GSLs (PRO and GCC) and *BrMYB51.1* was also positively correlated with total indolic GSLs and individual indolic GSLs (GBS and NGBS) in 38 accessions (Table 4). However, some TFs, such as *BrMYB28.2* for indolic GSL 4‐MOGBS and *BrMYB122.2* for aliphatic GSL SNG, showed contrasting regulatory responses. The differences in the regulation of *BrMYB* TFs between *B. rapa* and *A. thaliana* suggest that different mechanisms may operate in the genotype‐specific GSL biosynthesis pathway of *B. rapa*. GSL concentrations can be influenced by various factors, such as genotype differences, pre‐harvest conditions, cultural practices, stage of maturity and harvesting methods, as well as the interactions among these factors 20. This finding indicates that changes in genotype‐specific expression within *BrMYB* TFs are potentially correlated with phenotypic effects in polyploid *B. rapa*. Phenotypic effects of duplicated genes in polyploids have been reported previously for genes involved in regulating flowering time in *Arabidopsis* allotetraploids 39. Additionally, organ‐specific expression changes in duplicated genes have been demonstrated in the allopolyploid *Gossypium hirsutum* and *Brassica juncea* 40, 41. Thus, the divergence of paralogous genes may result in a functional alteration during the evolution of flowering plants.

Correlation analysis of the relative expression levels of *BrMYB* TFs related to GSL biosynthesis and GSL content in 38 accessions of *B. rapa* showed insufficient correlation with the content of both total and 12 identified GSLs. MYB TFs are known to be regulators of GSL biosynthesis genes, such as *AOP2*,* GSL‐OH* and *ST5*, etc. 12, 15, 41. Such an insufficient correlation between the expression level of *BrMYB* TFs and the content of total and aliphatic GSLs suggested the possibility that variation in the expression levels of GSL biosynthesis genes might lead to the changes in GSL content. Therefore, accurate observation of the expression levels of various GSL biosynthesis genes in 38 accessions will be required to determine the GSL content change by the expression network of the GSL biosynthesis genes involved with the 13 *BrMYB* TFs. Furthermore, functional studies are required to confirm that these *BrMYB* TFs are key regulators for GSLs biosynthesis in *B. rapa*.

Glucosinolates are known plant defense compounds in the Brassicaceae family 42. Transcript levels for some genes related to indolic and aliphatic GSL biosynthesis have been shown to increase resistance to various stresses, such as pathogens, insects, bacteria and herbivory in *Arabidopsis* 21, 43, 44. In the present study, all nine *BrMYB* TFs related to GSL biosynthesis showed increased expression levels in various stress treatments. The present study highlights the strong potential of these nine *BrMYB* TFs as regulators for various stress mechanisms. A functional approach to expression analysis of the *BrMYB* TFs and stress treatments on accessions with various GSL contents will help clarify the role of *BrMYB* TFs or GSLs for stress resistance mechanisms in *B. rapa*.

The present study improves our understanding of 13 *BrMYB* TFs duplicated by polyploid evolution in *B. rapa*. Our findings reveal that several *BrMYB* TFs are important for GSL biosynthesis and stress resistance mechanisms in *B. rapa*. Furthermore, the 38 *Brassica* accessions provide genetic diversity (through gene expression variation) and phenotypic diversity (in GSL content) for molecular breeding strategies in *B. rapa* and related species.

## Author contributions

JSK and MSS wrote the manuscript and carried out the experimental analysis. MJ and SHS were responsible for plant husbandry in the greenhouse and for the analysis of the traits of the 38 accessions.

## Supporting information


**Table S1.** List of primers used for real‐time PCR.
**Table S2.** Correlation analysis of expression level of *BrMYB* TFs related to GSL biosynthesis and GSL content in 19 accessions of *Brassica rapa* ssp. *pekinensis*.
**Table S3.** Correlation analysis of expression level of *BrMYB* TFs related to GSL biosynthesis and GSL content in five accessions of *Brassica rapa* ssp. *chinensis*.
**Table S4.** Correlation analysis of expression level of *BrMYB* TFs related to GSL biosynthesis and GSL content in six accessions of *Brassica rapa* ssp. *rapa*.
