# Supplementary material for: Expression profiles of BrMYB transcription factors related to glucosinolate biosynthesis and stress response in eight subspecies of Brassica rapa
Source: FEBS Open Bio. 2017 Sep 19;7(11):1646–59. doi: 10.1002/2211-5463.12231 (PMC5666390; doi:10.1002/2211-5463.12231)
Supplement: Supplementary file 1 — Table S1. List of primers used for real‐time PCR. Table S2. Correlation analysis of expression level of BrMYB TFs related to GSL biosynthesis and GSL content in 19 accessions of Brassica rapa ssp. pekinensis. Table S3. Correlation analysis of expression level of BrMYB TFs related to GSL biosynthesis and GSL content in five accessions of Brassica rapa ssp. chinensis. Table S4. Correlation analysis of expression level of BrMYB TFs related to GSL biosynthesis and GSL content in six accessions of Brassica rapa ssp. rapa. [file FEB4-7-1646-s001.pdf]

Table S1. List of primers used for real-time PCR.

Gene name		Primer sequence
BrMYB28.1	F	5´-TTCCTGACAAATACTCTCGCC-3´
	R	5´-CCGAGATCAGAAGTAGCGTTG-3´
BrMYB28.2	F	5´-GGTTTCAACACGGTTTTCGAG-3´
	R	5´-AAGCCTTGATGTAGAACTTGATTTC-3´
BrMYB28.3	F	5´-CCTGGGAACTTGCATTCTCTAG-3´
	R	5´-GAAAGAGGACGAGACATGGAAG-3´
BrMYB29.1	F	5´-GAACCTTGATCCCCTCTACAAC-3´
	R	5´-ATTGCTCCAGAAACTCCGAG-3´
BrMYB34.1	F	5´-GGATCATGTTGGTCTTATGGAGG-3´
	R	5´-CTCCAAGTCCAGATCGTCATTC-3´
BrMYB34.2	F	5´-ACCAAGAATTCGACCTCCAC-3´
	R	5´-AGAACAGTTGTCGGAGAAACC-3´
BrMYB34.3	F	5´-GAACATTCAACGTCCACACTG-3´
	R	5´-GAAAGAACCGGGAGAAGTCG-3´
BrMYB34.4	F	5´-TGCATTATGGAAGAGTTGAGGG-3´
	R	5´-AGAACCAAGAAACTCCATCCC-3´
BrMYB51.1	F	5´-ATTTGCAGGATATTCTGGAG-3´
	R	5´-GAACCTAGCCGACTTCTCCATCTT-3´
BrMYB51.2	F	5´-TCTTTCACACCAACCTCAGATC-3´
	R	5´-ATCATTAACGGATGCCTCAGAG-3´
BrMYB51.3	F	5´-ACAGGGTGGCTAATAGGTTTG-3´
	R	5´-ACTTATCTGAAATAGAGTCAACGGTG-3´
BrMYB122.1	F	5´-CTCTCCACACCAAACTCTTCG-3´
	R	5´-TGAGCGGAGGTATCGTTAGAG-3´
BrMYB122.2	F	5´-ACAAATCACTTAATGGTAAATCA-3´
	R	5´-AGAACCGAGCTGATAATGATCC-3´
BrActin	F	5´-GAACCGGGTGCTCCTCAGGA-3´
	R	5´-ATGGTACCGGAATGGTCAAGGC-3´

Table S2. Correlation analysis of relative expression level of *BrMYB* TFs related to GSL biosynthesis and GSL content in 19 accessions of *B. rapa* ssp. *pekinensis*.

Gene	PRO	GRA	SNG	GAL	GNL	GNA	GCC	GBN	Aliphatic GSLs	GBS	4-MOGBS	NGBS	Indolic GSLs	GNT	Total GSLs
BrMYB28.1	-0.183	-0.185	-0.163	-0.092	-0.229	-0.108	-0.080	-0.149	-0.149	-0.146	0.106	-0.283	-0.150	-0.111	-0.178
BrMYB28.2	-0.186	-0.208	0.221	-0.377	-0.234	-0.131	-0.103	-0.087	-0.164	0.139	0.251	-0.331	0.038	-0.053	-0.147
BrMYB28.3	0.214	-0.208	0.433	-0.027	-0.111	0.352	0.294	**0.530***	0.428	-0.133	-0.093	-0.346	-0.243	**0.466***	0.392
BrMYB29.1	0.154	-0.074	**0.495***	-0.239	-0.035	-0.128	-0.085	-0.062	-0.112	0.184	0.101	-0.262	0.032	0.192	-0.088
BrMYB34.1	-0.007	0.097	0.022	-0.273	-0.008	-0.091	-0.291	0.059	-0.078	0.079	0.009	-0.095	0.008	-0.036	-0.077
BrMYB34.2	-0.144	-0.168	-0.004	-0.291	-0.110	-0.197	-0.371	-0.231	-0.259	-0.344	-0.277	-0.395	-0.445	-0.352	-0.349
BrMYB34.3	-0.335	-0.267	-0.005	-0.364	-0.339	-0.235	-0.415	-0.254	-0.318	-0.141	-0.301	-0.429	-0.360	-0.372	-0.387
BrMYB34.4	-0.176	-0.076	-0.021	-0.104	-0.135	-0.170	-0.227	-0.217	-0.217	-0.468	-0.222	-0.208	-0.416	-0.251	-0.297
BrMYB51.1	-0.112	0.347	-0.146	0.390	-0.098	-0.134	-0.123	-0.147	-0.124	**0.590****	0.370	**0.488***	**0.648****	-0.001	-0.005
BrMYB51.2	0.403	0.058	-0.101	-0.155	**0.619****	0.028	-0.177	0.103	0.070	-0.180	-0.296	0.000	-0.208	0.018	0.029
BrMYB51.3	-0.214	-0.216	-0.160	-0.126	-0.253	-0.059	-0.006	-0.114	-0.106	-0.056	0.188	-0.269	-0.065	-0.094	-0.120
BrMYB122.1	-0.248	-0.181	0.304	-0.238	-0.159	-0.191	-0.236	-0.263	-0.260	0.113	-0.020	-0.346	-0.085	-0.149	-0.266
BrMYB122.2	-0.270	-0.104	**0.551***	-0.248	-0.318	0.048	0.052	-0.014	-0.011	0.288	0.079	-0.099	0.144	0.120	0.034

* and ** indicated a significant correlated at the level of $P < 0.05$, $P < 0.01$, respectively and without any * means not significant.

Table S3. Correlation analysis of relative expression level of *BrMYB* TFs related to GSL biosynthesis and GSL content in five accessions of *B. rapa* ssp. *chinensis*.

Gene	PRO	GRA	SNG	GAL	GNL	GNA	GCC	GBN	Aliphatic GSLs	GBS	4-MOGBS	NGBS	Indolic GSLs	GNT	Total GSLs
BrMYB28.1	-0.414	0.552	0.079	-0.480	-0.514	-0.298	-0.311	-0.751	-0.731	-0.531	0.826	-0.353	-0.271	-0.542	-0.688
BrMYB28.2	-0.111	0.433	0.429	-0.690	-0.129	-0.345	-0.377	-0.923	-0.788	-0.870	0.470	-0.705	-0.833	-0.940	-0.856
BrMYB28.3	**0.928***	-0.805	-0.434	0.648	0.815	-0.789	0.373	0.405	-0.353	0.277	-0.056	-0.680	-0.137	-0.231	-0.346
BrMYB29.1	-0.128	0.402	-0.384	-0.187	-0.249	-0.572	-0.366	-0.431	-0.800	-0.305	**0.977****	-0.319	-0.026	-0.320	-0.701
BrMYB34.1	-0.405	0.722	-0.415	-0.352	-0.423	-0.184	-0.641	-0.331	-0.466	-0.277	**0.905***	0.205	0.222	0.069	-0.356
BrMYB34.2	-0.478	0.767	-0.237	-0.426	-0.337	0.361	-0.732	-0.057	0.156	-0.171	0.335	0.713	0.337	0.474	0.211
BrMYB34.3	-0.314	0.679	-0.445	-0.338	-0.344	-0.301	-0.646	-0.355	-0.570	-0.315	**0.949***	0.085	0.151	-0.020	-0.463
BrMYB34.4	-0.622	**0.968****	-0.004	-0.723	-0.509	0.260	-0.828	-0.490	-0.167	-0.520	0.557	0.430	0.015	0.069	-0.136
BrMYB51.1	-0.370	-0.193	-0.192	0.514	-0.399	0.586	0.471	0.655	0.807	0.823	-0.320	0.824	**0.910***	**0.902***	**0.893***
BrMYB51.2	-0.172	0.435	-0.363	-0.213	-0.289	-0.535	-0.377	-0.448	-0.782	-0.314	**0.978****	-0.288	-0.017	-0.309	-0.684
BrMYB51.3	**0.973****	-0.545	-0.593	0.465	**0.978****	-0.711	-0.071	0.478	-0.273	0.143	-0.047	-0.425	-0.112	-0.037	-0.272
BrMYB122.1	-0.498	0.789	-0.152	-0.485	-0.341	0.414	-0.744	-0.099	0.185	-0.222	0.275	0.711	0.275	0.439	0.223
BrMYB122.2	-0.305	0.273	**0.888***	-0.674	-0.281	0.172	-0.026	-0.834	-0.262	-0.714	-0.118	-0.483	-0.824	-0.815	-0.392

* and ** indicated a significant correlated at the level of P< 0.05, P<0.01, respectively and without any * means not significant.

Table S4. Correlation analysis of relative expression level of *BrMYB* TFs related to GSL biosynthesis and GSLs content in six accessions of *B. rapa* ssp. *rapa*.

Gene	PRO	GRA	SNG	GAL	GNL	GNA	GCC	GBN	Aliphatic GSLs	GBS	4-MOGBS	NGBS	Indolic GSLs	GNT	Total GSLs
BrMYB28.1	0.428	-0.141	-0.064	-0.102	-0.137	0.612	0.310	-0.184	0.664	-0.513	0.165	-0.763	-0.701	-0.367	0.633
BrMYB28.2	0.514	-0.103	-0.726	-0.670	0.600	-0.337	0.765	-0.236	-0.153	0.108	0.114	-0.523	-0.066	-0.924	-0.168
BrMYB28.3	-0.243	-0.444	-0.782	-0.520	**0.876***	-0.893	0.564	0.289	-0.630	0.506	-0.066	0.316	0.555	-0.310	-0.614
BrMYB29.1	0.657	0.481	0.462	0.505	-0.285	0.466	-0.476	0.218	0.431	0.424	-0.518	0.568	0.465	0.251	0.456
BrMYB34.1	0.133	-0.287	-0.909	-0.412	0.627	-0.404	**0.990****	-0.239	-0.155	-0.231	0.452	-0.540	-0.296	-0.866	-0.180
BrMYB34.2	-0.010	-0.467	-0.706	-0.425	0.431	-0.123	**0.844***	-0.183	0.082	-0.455	0.449	-0.740	-0.572	-0.655	0.050
BrMYB34.3	-0.119	-0.731	0.015	-0.338	0.434	-0.027	-0.238	**0.954****	0.188	0.697	-0.821	0.618	0.657	0.570	0.224
BrMYB34.4	-0.122	0.024	0.244	**0.837***	-0.320	0.457	-0.165	0.232	0.498	-0.275	0.064	0.375	-0.096	0.453	0.502
BrMYB51.1	-0.730	0.435	0.235	0.228	-0.453	-0.369	-0.217	-0.656	-0.685	-0.496	0.688	-0.187	-0.350	0.110	-0.701
BrMYB51.2	-0.403	-0.035	0.325	0.549	-0.119	-0.032	-0.481	0.486	-0.032	0.298	-0.287	**0.873***	0.519	0.752	0.000
BrMYB51.3	0.216	-0.208	-0.812	-0.439	0.508	-0.283	**0.935****	-0.345	-0.087	-0.315	0.475	-0.694	-0.422	-0.904	-0.117
BrMYB122.1	-0.133	-0.561	0.300	-0.413	-0.109	0.429	-0.281	0.354	0.420	-0.063	-0.350	-0.304	-0.252	0.382	0.416
BrMYB122.2	-0.302	-0.128	0.310	0.757	-0.331	0.450	-0.258	0.331	0.479	-0.275	0.013	0.384	-0.105	0.615	0.485

* and ** indicated a significant correlated at the level of P< 0.05, P<0.01, respectively and without any * means not significant.
